# Theoretical Analysis and Structural Optimization of Overload-Protected MEMS Hydrophones

**DOI:** 10.3390/mi17040500

**Published:** 2026-04-20

**Authors:** Yuhan Ren, Jinming Ti, Qingqing Fan, Yanfeng Huang, Junhong Li

**Affiliations:** 1 State Key Laboratory of Acoustics and Marine Information, Institute of Acoustics, Chinese Academy of Sciences, Beijing 100190, China; 2 University of Chinese Academy of Sciences, Beijing 100049, China

**Keywords:** MEMS, hydrophone, overload-protection design, buckling stability

## Abstract

MEMS hydrophones, as critical sensors for maritime security and underwater information acquisition, have sensitive membrane structures that exhibit insufficient ability to withstand hydrostatic pressure, necessitating an overload-protection design. Based on buckling stability theory, a collaborative optimization method for overload-protection column design was proposed, integrating theoretical analysis, finite-element simulation, and process feasibility. An optimized design scheme for hydrophone overload-protection columns was established by comprehensively considering geometric buckling-resistant design, micro-gap anti-adhesion requirements, minimal impact on sensitivity, and micro/nano-fabrication constraints. The results indicate that intermediate slenderness columns with radii between 5.5 μm and 7.5 μm sufficiently meet both fabrication and operational requirements, effectively providing overload protection. Furthermore, at water depths not exceeding 382 m, the MEMS hydrophone can maintain the integrity of its membrane structure without column buckling.

## 1. Introduction

**Figure 1:**
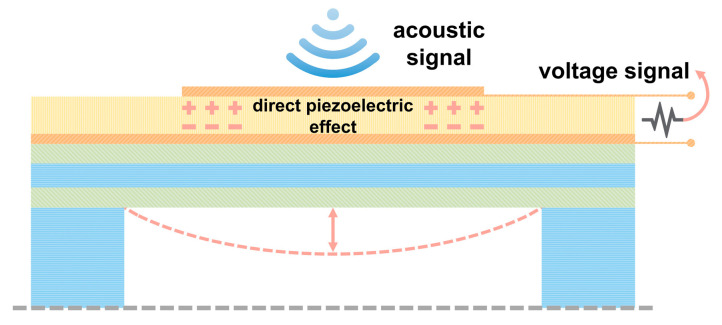
Schematic of the operating principle of a MEMS piezoelectric hydrophone.

MEMS hydrophones, with advantages such as miniaturization, array capability, and high sensitivity [1], have become one of the current research focuses in the field of acoustic sensing [2]. They show broad application prospects in areas including maritime security [3,4,5], noise monitoring [6,7,8,9], resource exploration [3], and pipeline leakage detection [6,10,11]. The sensing element of a MEMS hydrophone is a vibrating membrane that undergoes bending deformation when subjected to acoustic pressure. Due to the piezoelectric effect, induced charges are generated on the surface of the piezoelectric layer, enabling the detection of acoustic signals. The working principle of MEMS piezoelectric hydrophones is illustrated in Figure 1. To achieve high sensitivity, the back cavity of the membrane is typically air- or vacuum-filled [12]. However, in underwater environments, the membrane is prone to failure or rupture due to excessive hydrostatic pressure.

Three main structural optimization approaches have been developed to address the aforementioned issue. The most straightforward method is to completely fill the back cavity with a liquid such as castor oil [13], which suppresses excessive membrane deformation but significantly reduces the hydrophone sensitivity. Choi et al. [12,14] and Yang et al. [15] proposed integrating a pressure-balancing module with microchannels and microcavities into the back cavity. In this design, liquid enters the microchannels and compresses the air in the cavity, thereby equalizing the pressure on both sides of the membrane. This approach maintains the air-backed configuration and preserves high sensitivity. However, fabricating the microchannels is challenging, and their length must be carefully optimized—excessive length drastically lowers the chip area utilization, whereas insufficient length weakens the pressure-balancing effect. Xu et al. [16,17] introduced multiple supporting structures within the back cavity to sustain the excessively deflected membrane. This design not only ensures efficient utilization of the chip area but also retains the high sensitivity of air-backed hydrophones. Considering these limitations, this study proposes a MEMS hydrophone incorporating overload-protection columns. A relatively thick membrane is adopted to withstand hydrostatic pressure, while the columns within the back cavity provide additional support against excessive deflection, thereby enhancing the overall ability to withstand hydrostatic pressure. Since no systematic theoretical guidance or methodological framework currently exists for the design of overload-protection columns in MEMS hydrophones, a comprehensive design strategy combining theoretical analysis, finite-element simulation, and process feasibility evaluation is proposed, offering a new pathway and technical reference for MEMS hydrophone performance optimization.

## 2. Theoretical Analysis of MEMS Hydrophone with Overload-Protection Column

The overload-protection column supports the membrane when it bends excessively under high hydrostatic pressure. It prevents plastic deformation or cracking, thereby avoiding irreversible damage to the hydrophone if the operating depth is accidentally exceeded. This design significantly improves the reliability and service life of the hydrophone. The column is analyzed using a fixed-free buckling model. Columns with excessive slenderness are prone to structural instability, whereas columns with low slenderness occupy a large fraction of the cavity volume, leading to degradation of hydrophone sensitivity. Therefore, the theoretical analysis of the overload-protection column is conducted from two key perspectives: the geometric stability of the column structure and impact of introducing the overload-protection column on hydrophone sensitivity.

### 2.1. Structural Design of MEMS Hydrophone with Overload-Protection Column

**Figure 2:**
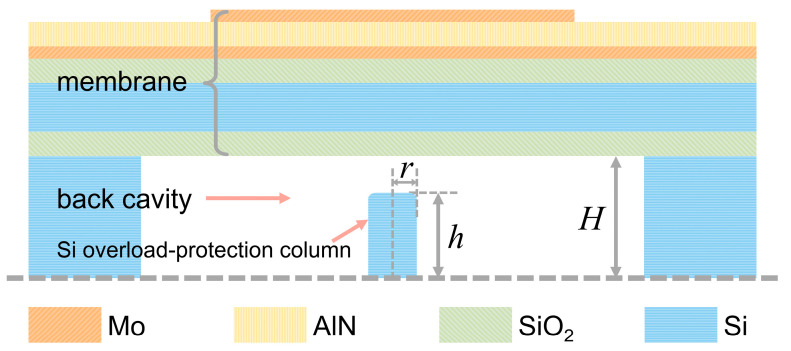
Schematic cross-sectional view of the MEMS hydrophone with overload-protection columns.

The structure of the MEMS hydrophone with overload-protection columns is shown in Figure 2. The hydrophone mainly consists of three components: a vibrating membrane, a back cavity, and overload-protection columns. Under incident acoustic pressure, the vibrating membrane undergoes bending deformation, which induces electric charges on the AlN piezoelectric layer via the piezoelectric effect, thereby enabling acoustic signal detection. The back cavity is designed as a sealed air cavity, which offers superior sensitivity compared with an oil-filled back cavity. The overload-protection columns are located within the cavity and serve to protect the device under overload conditions.

The primary structural parameters influencing the performance of the overload-protection columns include the cavity height *H*, column height *h*, and column radius *r*. In the design process, these parameters were systematically varied to analyze how membrane deformation under the same hydrostatic pressure influences the stress distribution in columns of different dimensions. The objective is to develop an overload-protection column structure that can provide effective mechanical support for the membrane under overload conditions while having minimal impact on the hydrophone’s original performance once the overload is removed.

### 2.2. Buckling Stability Analysis

The overload-protection column can be geometrically simplified as a cylindrical column with one end fixed and the other end free. The slenderness ratio of the column is defined as

(1) $$\lambda =\frac{\mu h}{i},$$

where *μ* is the effective length coefficient determined by the boundary conditions at both ends of the column (for a fixed-free column, μ=2); $i=\sqrt{I/A}$ is the radius of gyration; *I* is the area moment of inertia of the column cross-section (for a circular cross-section, $I=\pi r^{4}/4$); and *A* is the cross-sectional area. Therefore, Equation (1) can be simplified as

(2) $$\lambda =\frac{4h}{r}.$$

**Figure 3:**
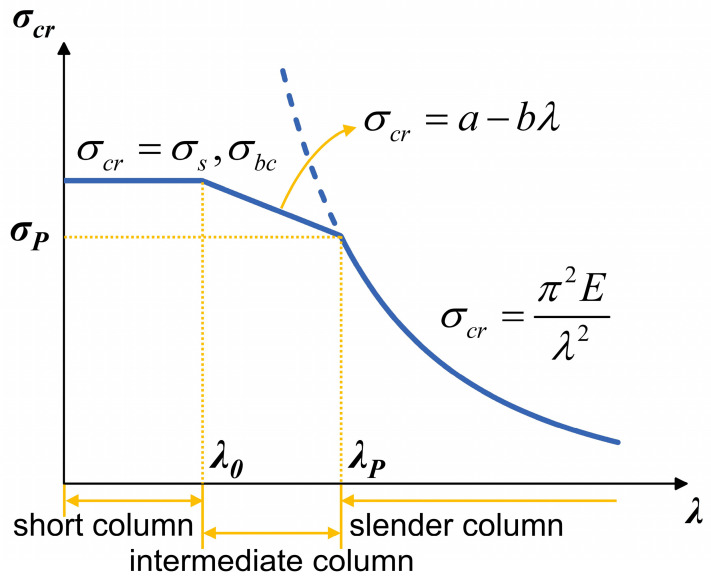
Relationship between theoretical critical stress *σ_cr_* and slenderness ratio *λ* of the column.

Buckling occurs when the axial stress of the column exceeds its critical stress *σ_cr_*, leading to structural instability and potential failure. The relationship between the theoretical critical stress *σ_cr_* and the slenderness ratio *λ* is shown in Figure 3. The corresponding analytical expression can be represented as a continuous piecewise function with respect to *λ*:

(3) $$\sigma_{cr}=\left\{\begin{array}{cc} \pi^{2}E/\lambda^{2} & \left(\lambda \geq \lambda_{P}\right)\\ a-b\lambda & \left(\lambda_{0}\leq \lambda <\lambda_{P}\right)\\ \sigma_{0} & \left(0\leq \lambda <\lambda_{0}\right) \end{array}\right.,$$

For slender columns ($\lambda \geq \lambda_{P}$), the critical stress *σ_cr_* is calculated using Euler’s formula, where *E* is the elastic modulus of the column, $\lambda_{P}=\pi \sqrt{E/\sigma_{P}}$, and *σ_P_* is the proportional limit of the material. For short and intermediate columns ($0\leq \lambda <\lambda_{P}$), Euler’s formula is no longer applicable. Instead, a linear empirical formula is used to determine *σ_cr_*, where *a* and *b* are material constants, usually obtained from tables. For plastic materials, *σ*_0_ is taken as the yield strength *σ_s_*, whereas for brittle materials, *σ*_0_ is taken as the ultimate compressive strength *σ_bc_*. In Equation (3), $\lambda_{0}=\left(a-\sigma_{0}\right)/b$.

### 2.3. Pressure Variation in the Back Cavity

**Figure 4:**
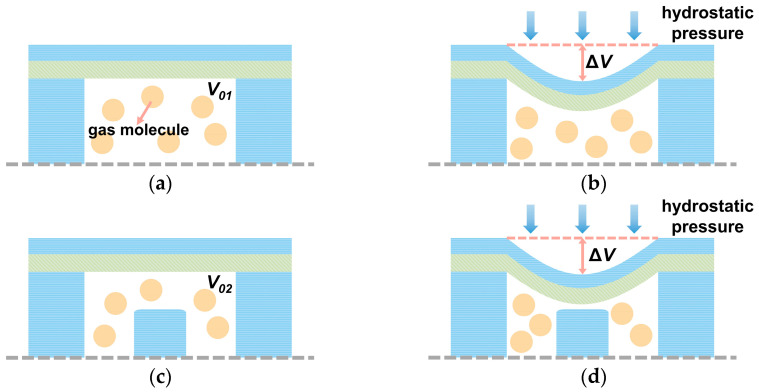
Schematic illustration of the effect of overload-protection column on back cavity pressure under hydrostatic pressure: (**a**) without column, no hydrostatic pressure; (**b**) without column, with hydrostatic pressure; (**c**) with column, no hydrostatic pressure; (**d**) with column, with hydrostatic pressure.

Figure 4 shows the variation in back cavity pressure under hydrostatic pressure with and without overload-protection column. As shown in Figure 4a,c, when the cavity pressure is identical, the presence of overload-protection column reduces the effective volume of the cavity gas (*V*_0_), resulting in $V_{01}>V_{02}$. Under hydrostatic pressure, the membrane deflects inward, compressing the cavity gas and increasing the cavity pressure. For the same hydrostatic pressure, the membrane deflection and the corresponding volume change (Δ*V*) can be assumed identical. As shown in Figure 4b,d, a larger pressure increase is observed in the cavity with overload-protection column. Consequently, columns with different geometrical dimensions lead to different pressure increases in the back cavity. This elevated pressure suppresses the effective vibration of the membrane and reduces the sensitivity of the hydrophone. Therefore, the influence of geometric parameters on back cavity pressure should be investigated.

Assuming there are *m* overload-protection columns within the back cavity and that the membrane has a radius *R*, the initial volume of the cavity gas can be expressed as

(4) $$V_{0}=\pi R^{2}H-m\pi r^{2}h.$$

After compression, the gas volume *V*_1_ becomes $V_{1}=V_{0}-∆V$, and the relationship between the initial pressure *P*_0_ and the compressed pressure *P*_1_ satisfies ${P_{0}V}_{0}=P_{1}V_{1}$. Thus, *P*_1_ can be expressed as

(5) $$P_{1}=P_{0}\left[1+\frac{\Delta V}{\pi R^{2}H-m\pi r^{2}h-\Delta V}\right],$$

the cavity height *H* and the column height *h* satisfy the relation h=H−x, where *x* represents the maximum displacement of the membrane bottom surface at its rated operating depth and can be regarded as a fixed value. Therefore, Equation (5) can also be rewritten as

(6) $$P_{1}=P_{0}\left[1+\frac{\Delta V}{(\pi R^{2}-m\pi r^{2})H+m\pi r^{2}x-\Delta V}\right].$$

Combining Equations (5) and (6), it can be concluded that a smaller column radius *r* or a larger cavity height *H* results in a lower compressed cavity pressure *P*_1_, thereby reducing the restriction on membrane vibration and improving the hydrophone’s sensitivity.

### 2.4. Squeeze-Film Damping Analysis of the Membrane Back Cavity

**Figure 5:**
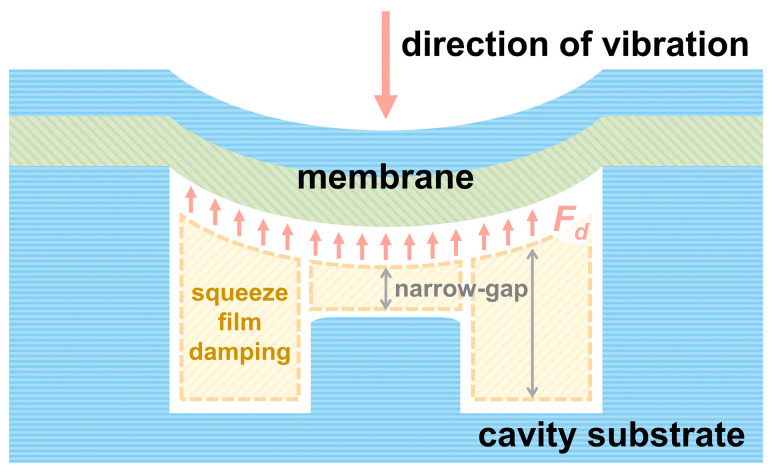
Schematic illustration of squeeze-film damping.

The gaps between the membrane and the overload-protection columns, as well as between the membrane and the cavity substrate, are on the micrometer scale, forming a typical narrow-gap structure. When the membrane undergoes periodic vibration under acoustic pressure, the gas within these narrow gaps is periodically compressed and expanded. Due to the small gap height, gas flow is highly restricted. Accordingly, viscous resistance is generated in the gas layer, leading to squeeze-film damping [18], as illustrated in Figure 5. This damping effect causes energy dissipation during vibration, reduces the effective vibration amplitude of the membrane, and consequently decreases the sensitivity of the hydrophone and increases the noise level. Therefore, it is necessary to investigate how the key geometric parameters influence squeeze-film damping.

The squeeze-film damping coefficient *c* (unit: N·s/m) between a circular membrane and the substrate [19] is expressed as

(7) $$c=\frac{3\eta S^{2}}{2\pi {h_{0}}^{3}},$$

where *η* is the gas viscosity, *S* is the effective area between the membrane and the substrate, and *h*_0_ is the gap height.

For the membrane–substrate gap, $h_{0}=H$, while for the gap between the membrane and the column top, $h_{0}=x$. The total squeeze-film damping of a sealed back cavity with overload-protection columns can therefore be obtained using the partition integration method:

(8) $$c=c_{1}+c_{2}=\frac{3\pi \eta }{2}\cdot \left(\frac{{\left(mr^{2}\right)}^{2}}{x^{3}}+\frac{{\left(R^{2}-mr^{2}\right)}^{2}}{H^{3}}\right).$$

For small-amplitude vibrations, the damping force *F_d_* is given by

(9) $$F_{d}=-cv,$$

where *v* denotes the membrane vibration velocity (unit: m/s).

A larger damping coefficient *c* results in improved transient response but faster attenuation of high-frequency signals, thereby limiting the hydrophone’s bandwidth [20]. Excessive damping force also suppresses the membrane amplitude, reducing the hydrophone’s sensitivity.

The mechanical quality factor *Q* of the system under damped vibration [21] is defined as

(10) $$Q=\frac{\omega_{0}M}{c},$$

where *ω*_0_ is the resonant angular frequency of the membrane and *M* is the membrane mass.

When *c* is small, the damping force *F_d_* exerts less suppression on the membrane amplitude, resulting in higher sensitivity. However, an excessively high *Q* value produces an overly sharp resonance peak, making the response more susceptible to environmental disturbances and signal distortion.

Since the hydrophone operates primarily in the flat frequency-response region far from resonance, slight distortion near the resonance frequency does not significantly affect device performance. Therefore, structural design should aim to avoid high squeeze-film damping to ensure optimal hydrophone sensitivity.

## 3. Simulation Design and Analysis of the Membrane with Overload-Protection Column

### 3.1. Finite-Element Analysis of the Membrane with Overload-Protection Column

As shown in Figure 2, aluminum nitride (AlN) was selected as the piezoelectric layer due to its excellent compatibility with MEMS fabrication processes. Molybdenum (Mo) was used for the top and bottom electrodes. The lower layers of the membrane consist of a silicon dioxide (SiO_2_) layer, a device silicon layer, and a thermally oxidized SiO_2_ layer, which together enhance the bending stiffness of the membrane. Single-crystal silicon (Si) was chosen as the material for the overload-protection columns.

In the subsequent analysis, a single-column configuration was primarily considered, providing a reference for the optimization of multi-column structures. The material parameters used in the simulation are listed in Table 1.

For an *N*-layer composite membrane, the first-order resonant frequency *f*_0_ can be expressed as [23]

(11) $$f_{0}=\frac{5.104}{\pi R^{2}}\sqrt{\frac{D}{\rho_{s}}},$$

where *D* is the equivalent flexural rigidity of the composite membrane [24]:

(12) $$D=\frac{1}{3}\sum_{k=1}^{N}\frac{E_{k}}{1-\nu_{k}^{2}}[{\left(z_{k}-z^{0}\right)}^{3}-{\left(z_{k-1}-z^{0}\right)}^{3}].$$

Here, *E_k_*, *υ_k_*, *z_k_*, and *z_k_*_−1_ denote the Young’s modulus, Poisson’s ratio, and the coordinates of the upper and lower surfaces of the *k*-th layer, respectively. *z*^0^ represents the position of the neutral plane of the composite membrane, $z^{0}=\frac{\sum_{k=1}^{N}\frac{E_{k}}{1-\nu_{k}}t_{k}\frac{\left(z_{k}+z_{k-1}\right)}{2}}{\sum_{k-1}^{N}\frac{E_{k}}{1-\nu_{k}}t_{k}}$ [23], and *t_k_* is the thickness of the *k*-th layer. In Equation (11), *ρ_s_* is the areal density of the composite membrane, given by $\rho_{S}=\sum_{k=1}^{N}t_{k}\rho_{k}$, where *ρ_k_* is the density of the *k*-th layer.

**Figure 6:**
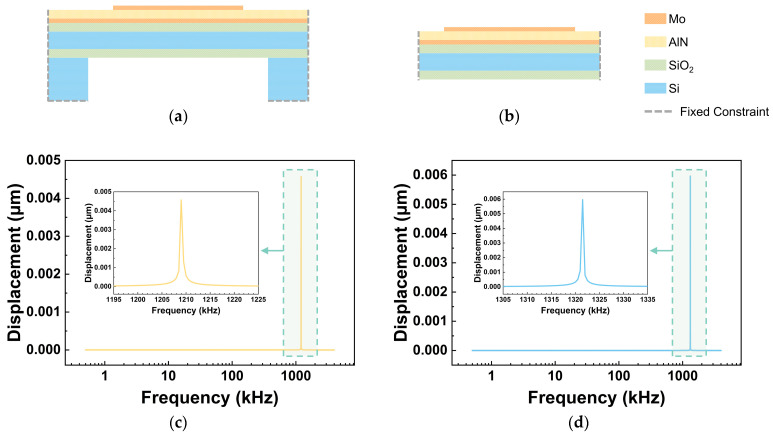
Membrane models and their frequency responses: (**a**,**c**) membrane with peripheral support structures and its frequency response; (**b**,**d**) model constructed according to the theoretical analysis and its frequency response.

Based on the material parameters listed in Table 1, the theoretical first-order resonant frequency *f*_0_ of the membrane is calculated to be 1336.7 kHz using Equation (11). The frequency response of the structure shown in Figure 6a is presented in Figure 6c, where the simulation result indicates a resonance peak near 1209.0 kHz. For comparison, a model constructed according to the theoretical analysis, illustrated in Figure 6b, yields the frequency response shown in Figure 6d, where a resonance peak appears at 1321.5 kHz, showing only a 1.14% deviation from the theoretical value. This consistency confirms the reliability of the finite-element simulation and demonstrates good agreement with the theoretical analysis.

### 3.2. Effect of Overload-Protection Column on the Stress of the Vibrating Membrane

To analyze the stress distribution of the vibrating membrane and the overload-protection column under overload conditions, the contact interaction between the two components should be established in the model. When the operating depth exceeds the rated range, the vibrating membrane deforms under pressure and contacts the overload-protection column at the cavity center, transmitting stress to the column below and causing it to undergo stress and strain. To accurately simulate this process, a contact pair is defined in the finite-element model. The contact pair consists of a source boundary and a target boundary. In this model, the bottom surface of the vibrating membrane is set as the source, while the top surface of the column is designated as the target. The outer corner of the column top is rounded and included as part of the target boundary to avoid direct contact between sharp corners and surfaces. The Augmented Lagrangian method is adopted for contact pressure calculation, which provides higher computational accuracy at the cost of acceptable computational effort.

The mesh division must balance accuracy and computational efficiency. A refined mesh is applied to critical regions such as the overload-protection column, while non-critical regions such as the silicon substrate use a coarser mesh. For surface-to-surface contact, the target boundary should have at least twice the mesh density of the source boundary.

**Figure 7:**
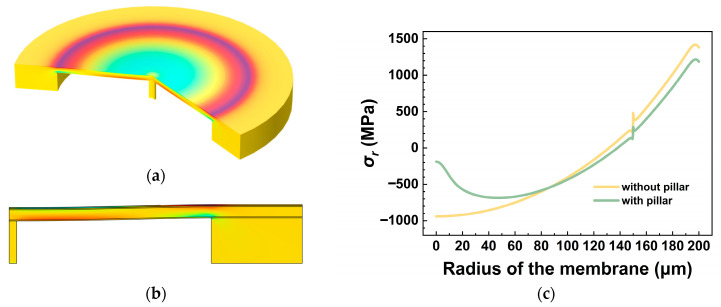
The stress distribution of the vibrating membrane with the overload-protection column under overload conditions: (**a**) 3D schematic diagram; (**b**) 2D cross-sectional view; (**c**) comparison of radial stress in the piezoelectric layer with and without the overload-protection column.

Figure 7 presents the stress distribution of the vibrating membrane and the comparison of radial stress in the piezoelectric layer with and without the overload-protection column under overload conditions. It can be observed that the stress in the contact area decreases once the membrane touches the overload-protection column, indicating that the column exerts an upward supporting force on the membrane—demonstrating the effectiveness of the support mechanism.

### 3.3. Effect of h–r Geometric Parameters on the Average Stress of the Overload-Protection Column

To investigate the overall mechanical behavior of the overload-protection column, this section analyzes the influence of the geometric parameters *h* and *r* on the average stress *σ_avg_* and elucidates the underlying physical mechanisms responsible for the observed trends.

**Figure 8:**
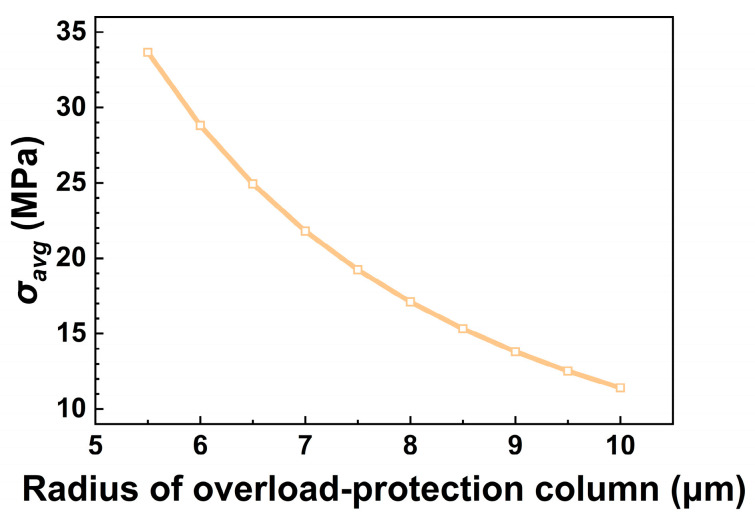
Variation in average stress *σ_avg_* with column radius *r* at h=42 μm.

The effect of column radius *r* on *σ_avg_* was first examined. Figure 8 presents the variation in *σ_avg_* as a function of *r* for a column height of h=42 μm.

The results indicate a negative correlation between *σ_avg_* and *r*, consistent with the trend predicted by the analytical stress formulation $\sigma =F/\pi r^{2}$. A larger radius results in lower average stress, suggesting that columns with greater radii possess enhanced load-distribution capability and higher load-bearing capacity.

**Figure 9:**
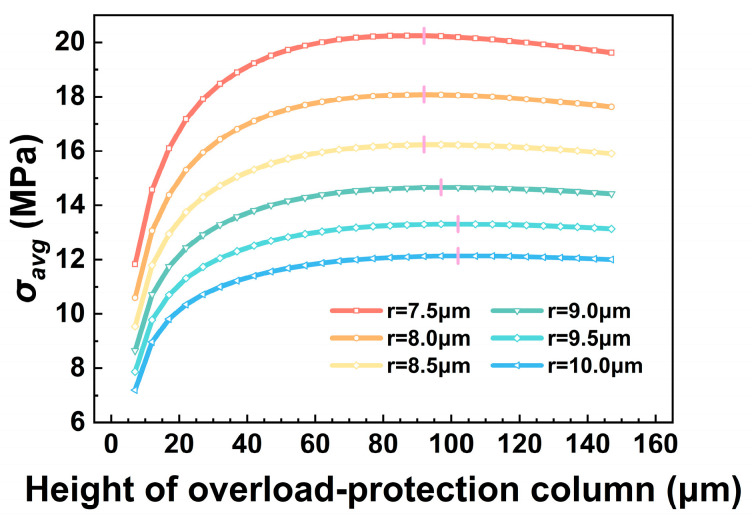
Variation in the average stress *σ_avg_* with column height *h* for overload-protection columns with different radii *r*.

Figure 9 shows the variation in *σ_avg_* with column height *h* under different radii *r*. It can be observed that *σ_avg_* reaches a maximum value as *h* increases, exhibiting an initial rising trend followed by a gradual decline. Moreover, *σ_avg_* varies more sharply at smaller *h* values, while the change becomes less pronounced as *h* continues to increase.

This trend is primarily attributed to the variation in the axial compressive stiffness *k_p_* of the overload-protection column with increasing height *h*.

When *h* is small, *k_p_* is relatively large, and the column provides an approximately rigid support to the vibrating membrane. Under this condition, the contact area between the membrane and the column is limited, resulting in pronounced local stress concentration. As *h* increases, *k_p_* decreases, making the column more susceptible to axial compression. Consequently, the contact area between the vibrating membrane and the column top gradually increases, and the stress-concentrated region at the column top expands accordingly. This expansion indicates an increase in the effective load-transfer area, leading to a continued increase in the average stress *σ_avg_*. When *k_p_* is reduced to a level comparable to the local stiffness *k_m_* of the vibrating membrane, *σ_avg_* reaches its maximum value.

With further increases in *h*, the overload-protection column becomes the relatively soft element in the contact pair. Larger axial compressive deformation then absorbs more energy, while the enlarged contact area between the membrane and the column top distributes the stress over a wider region. As a result, *σ_avg_* begins to decrease gradually.

**Figure 10:**
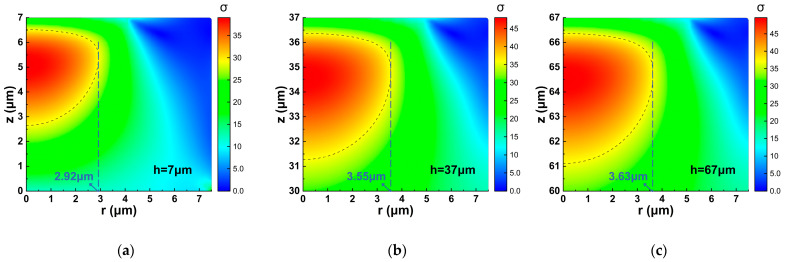
Schematic diagrams of stress distribution and stress concentration regions at the top of the overload-protection column for different heights *h*: (**a**) *h* = 7 μm; (**b**) *h* = 37 μm; (**c**) *h* = 67 μm.

Schematic illustrations of the stress concentration regions for columns with different heights are shown in Figure 10.

In addition, as the column radius *r* increases, the overall stiffness of the column increases, requiring a larger height *h* to satisfy the stiffness-matching condition. As a result, the location of the maximum *σ_avg_* shifts gradually toward higher *h* values with increasing *r*.

### 3.4. Effect of H–r Geometric Parameters on Hydrophone Sensitivity

**Figure 11:**
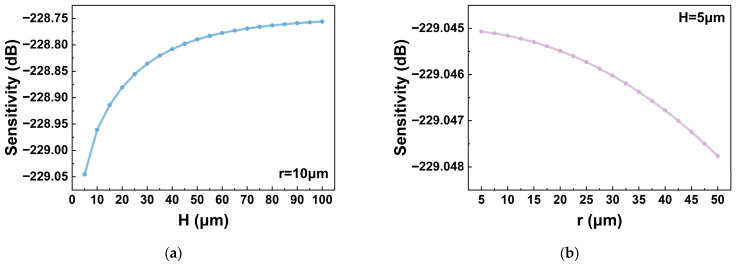
Variation in hydrophone sensitivity (@350 m, 200 Hz, ref. 1 V/μPa) with *H* and *r* considering only back cavity pressure: (**a**) effect of *H*; (**b**) effect of *r*.

To construct a geometrically sealed cavity structure, a silicon substrate layer was added to the model. The current cavity volume *V*_1_ was calculated using the divergence theorem. The corresponding cavity pressure *P*_1_ was then determined using the relation $P_{0}V_{0}=P_{1}V_{1}$, where *P*_0_ was set to the standard atmospheric pressure *P_atm_*, and the cavity medium was assumed to be air. The acoustic pressure loss introduced by the air medium was conservatively estimated at 1%. Figure 11 illustrates the variation in hydrophone sensitivity with respect to the parameters *H* and *r* at a water depth of 350 m, considering only the effect of back cavity pressure. As shown in Figure 11, in the presence of back cavity pressure, the sensitivity of the hydrophone can be effectively enhanced by increasing *H* and decreasing *r*. This observation is consistent with the analytical conclusions presented in Section 2.3.

**Figure 12:**
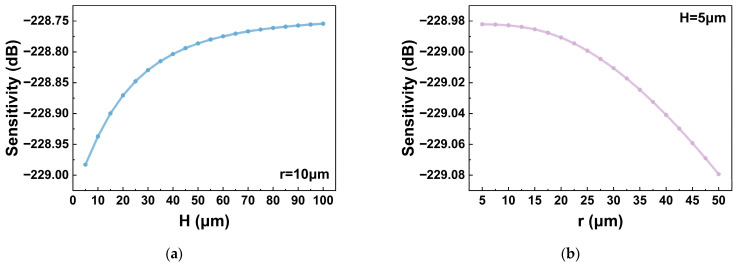
Variation in hydrophone sensitivity (@350 m, 200 Hz, ref. 1 V/μPa) with *H* and *r* considering only damping force: (**a**) effect of *H*; (**b**) effect of *r*.

Based on Equations (8) and (9), the distribution of damping force at the bottom surface of the vibrating membrane was calculated and subsequently applied to the membrane. Figure 12 presents the variation in hydrophone sensitivity as a function of *H* and *r* at a water depth of 350 m, considering only the effect of damping force. The results indicate that the hydrophone sensitivity is reduced when *H* is small and *r* is large, corresponding to strong membrane damping. This finding is consistent with the analysis presented in Section 2.4.

Overall, the effect of *r* on sensitivity is weaker than that of *H*, and damping force has a more pronounced influence than back cavity pressure. The finite-element results are consistent with the physical expectations presented in Section 2.3 and Section 2.4: decreasing *H* or increasing *r* reduces hydrophone sensitivity, indicating that columns with lower slenderness, corresponding to smaller *h* and larger *r*, degrade sensitivity. Proper selection of the *h*–*r* parameters therefore enables effective overload protection with minimal impact on sensitivity.

## 4. Design of the Overload-Protection Column Structure

In MEMS hydrophones, Si is commonly used to fabricate the cavity-supported overload-protection columns. However, as a brittle material, Si lacks widely accepted empirical constants *a* and *b*, making it difficult to directly apply Euler’s formula and the empirical formulas for geometric design.

**Figure 13:**
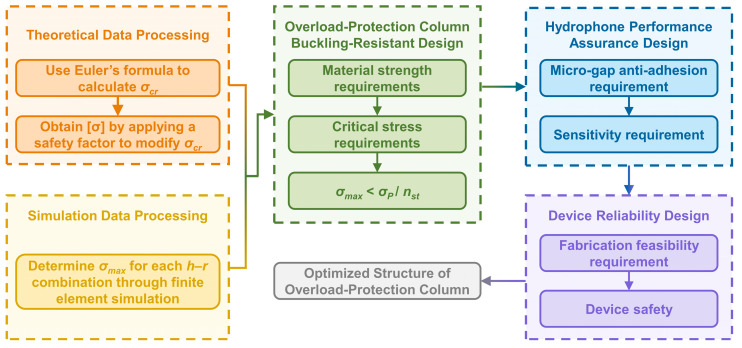
Optimized design scheme for the overload-protection column.

To overcome this limitation, an optimized structural design scheme for the overload-protection column is proposed in this study, based on the theoretical analysis of column buckling stability. The design framework comprehensively considers three aspects: geometric buckling resistance, minimization of adverse effects on hydrophone performance, and overall device reliability. The corresponding design process is illustrated in Figure 13.

To prevent membrane collapse caused by excessive deflection, the maximum deflection *x* of the vibrating membrane was limited to 3 μm, corresponding to a rated operating depth of approximately 350 m. A hydrostatic pressure equivalent to a depth of 375 m was applied to the membrane, causing it to contact the overload-protection column. The column height *h* and radius *r* were parametrically varied, and the maximum column stress *σ_max_* was obtained using the finite-element method (FEM).

For each combination of *h* and *r*, the slenderness ratio *λ* was calculated according to Equation (2). Based on these results, the columns were categorized into high, intermediate, and low slenderness regions. As Si exhibits no plastic deformation, its proportional limit *σ_P_* can be considered approximately equal to its ultimate tensile strength *σ_bt_*. Based on this, the design space was categorized into regions of high and low-to-intermediate slenderness. The theoretical critical stress *σ_cr-E_* for all *h*–*r* combinations was calculated using the Euler formula $\sigma_{cr-E}=\pi^{2}E/\lambda^{2}$. As shown in Figure 3, the Euler prediction is accurate for the high slenderness region, whereas in the low-to-intermediate region it tends to overestimate the true critical stress. The actual critical stress of the column is denoted as *σ_cr_*_0_, which satisfies

(13) $$\left\{\begin{array}{cc} \sigma_{cr0}=\sigma_{cr-E} & \left(\lambda \geq \lambda_{P}\right)\\ \sigma_{bt}\leq \sigma_{cr0}\leq \min\left(\sigma_{cr-E},\sigma_{bc}\right) & \left(\lambda_{0}\leq \lambda <\lambda_{P}\right)\\ \sigma_{cr0}=\sigma_{bc} & \left(0\leq \lambda <\lambda_{0}\right) \end{array}\right..$$

Here, *σ_bt_* and *σ_bc_* represent the ultimate tensile and compressive strengths of Si, respectively taken as 350 MPa and 950 MPa [25,26].

When the column stress reaches *σ_cr_*_0_, structural buckling occurs. Due to the brittle nature of Si, sudden fracture may take place; therefore, a relatively high safety factor *n_st_* must be applied to modify the Euler formula. In this study, $n_{st}=5$ [27] was adopted, leading to the allowable stress $\left[\sigma \right]=\sigma_{cr-E}/n_{st}$ and actual allowable stress $\left[\sigma_{0}\right]=\sigma_{cr0}/n_{st}$ for the overload-protection column, where $\left[\sigma \right]\geq \left[\sigma_{0}\right]$.

The buckling-resistant design strategy is to identify all *h*–*r* combinations that reliably satisfy $\sigma_{max}<\left[\sigma_{0}\right]$, thereby ensuring structural stability, by comparing the simulated stress *σ_max_* with the analytically calculated stress [σ]. The relationship between [σ] and the accurate allowable stress [σ_0_] ($\left[\sigma_{0}\right]\leq \left[\sigma \right]$) is used to indirectly assess the consistency between *σ_max_* and [σ_0_].

First, all *h*–*r* combinations were screened based on the compressive strength limit of the Si material. Combinations with $\sigma_{max}\geq \sigma_{bc}/n_{st}=190\;MPa$ were excluded, as they would result in strength failure of the overload-protection column.

Next, a critical stress-based screening was conducted. All combinations where $\sigma_{max}\geq \left[\sigma \right]$ were eliminated, since in these cases *σ_max_* would inevitably exceed [σ_0_], leading to buckling failure.

Finally, for the low-to-intermediate slenderness region, only the combinations satisfying $\sigma_{max}\leq \sigma_{bt}/n_{st}=70\;MPa$ were retained. Other combinations, for which the relationship between *σ_max_* and [σ_0_] could not be determined clearly, were conservatively discarded according to the principle of safe design.

Through this multi-stage screening process based on geometric configuration and stress analysis, the remaining *h*–*r* combinations were identified as meeting the criterion $\sigma_{max}\leq \left[\sigma_{0}\right]$. Finite-element simulations confirmed that these designs effectively prevent buckling in the overload-protection column.

Since interfacial adhesion is likely to occur within microscale gaps in MEMS devices, based on previous studies on anti-adhesion micro-bump structures [28,29], all cases with r>7.5 μm are excluded to ensure that the overload-protection columns satisfy the anti-adhesion requirement.

As discussed in Section 3.4, overload-protection columns with low slenderness are unfavorable for preserving hydrophone sensitivity. Accordingly, combinations with $\lambda <\lambda_{P}\times 30\%$ are regarded as low slenderness silicon columns and excluded from further consideration.

During the fabrication of MEMS devices, lateral etching can cause end thinning of high slenderness columns, substantially increasing the risk of column fracture. Therefore, high slenderness design combinations should be avoided to ensure fabrication feasibility.

**Figure 14:**
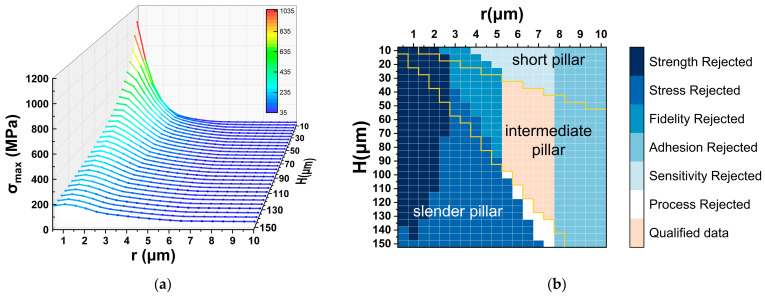
Stress distribution and optimized design results of the overload-protection column: (**a**) maximum stress *σ_max_* for various *h*–*r* dimensions; (**b**) data-filtering results based on the optimized design scheme.

Figure 14 illustrates the maximum stress *σ_max_* corresponding to all *h*–*r* parameter combinations, as well as the data-filtering process based on the optimized design criteria. The above analysis indicates that low slenderness columns tend to reduce sensitivity, while high slenderness columns are prone to buckling. When the column radius exceeds 7.5 μm, adhesion between the vibrating membrane and the overload-protection column occurs. As shown in Figure 14b, columns with intermediate slenderness, corresponding to radii between 5.5 μm and 7.5 μm, satisfy the requirements for buckling resistance, maintenance of sensitivity, and fabrication feasibility.

To further identify the optimal design, device safety was taken into consideration by selecting the *h*–*r* combination that provides the largest stress margin between [σ_0_] and *σ_max_*. This approach ensures that the maximum contact stress *σ_max_* remains well below the critical allowable stress [σ_0_] corresponding to structural buckling. For intermediate slenderness columns, the lower bound of [σ_0_] is conservatively set to 70 MPa. Following this procedure, the optimal design parameters are determined to be h=42 μm, r=7.5 μm. This result comprehensively considers theoretical analysis, simulation validation, and fabrication feasibility, providing the overload-protection column with a maximum safe operational margin while minimizing its impact on hydrophone sensitivity.

## 5. Simulation Analysis and Performance Verification

Due to the small contact area between the vibrating membrane and the overload-protection column, local stress concentration may occur in the supported region once contact takes place, potentially causing membrane fracture. Therefore, the stress distribution in each structural layer of the vibrating membrane under overload conditions needs to be analyzed. Furthermore, the pressure resistance of the hydrophone before and after structural optimization is evaluated, and the variation in hydrophone sensitivity under different hydrostatic pressures is investigated. In addition, the introduction of overload-protection columns reduces the effective cavity volume, making it necessary to analyze the sensitivity variation in the hydrophone under multiple-column configurations.

### 5.1. Stress Analysis of the Vibrating Membrane Layers

Based on the optimal design of the overload-protection column, the mechanical performance of the vibrating membrane was assessed to investigate its response under overload conditions. Finite-element simulations were conducted to analyze the mechanical behavior of each layer of the membrane at a water depth of 375 m, where the maximum radial stress *σ_rmax_* of each layer was obtained. The detailed results are summarized in Table 2.

According to Reference [12], when the maximum radial stress *σ_rmax_* exceeds approximately 1% of the Young’s modulus of the plate material, the thin plate reaches its deformation limit and fractures. As shown in Table 2, the simulated *σ_rmax_* values for all membrane layers remain below this limit, indicating that *σ_rmax_* stays within a safe stress range during contact with the overload-protection column, thereby preventing membrane failure.

### 5.2. Pressure Resistance Analysis of the Hydrophone

**Figure 15:**
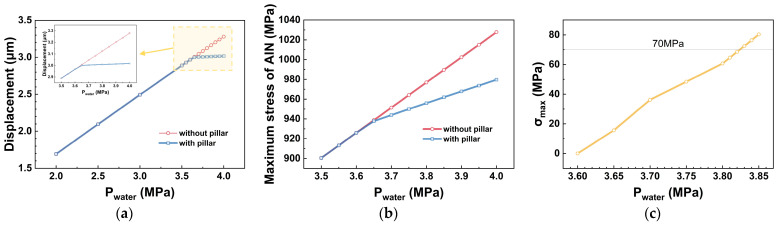
Variation in different parameters in the hydrophone with and without the overload-protection column under different hydrostatic pressures: (**a**) maximum displacement of the bottom surface; (**b**) maximum stress in the piezoelectric layer; (**c**) maximum stress *σ_max_* in the overload-protection column.

Figure 15 compares the pressure resistance performance of the hydrophone structure before and after optimization in terms of the maximum deflection of the vibrating membrane, the maximum stress in the piezoelectric layer, and the maximum stress in the overload-protection column. As shown in Figure 15a, when the water depth reaches 360 m, the vibrating membrane begins to make contact with the overload-protection column. As the hydrostatic pressure increases, the overload-protection column effectively limits the deformation of the membrane, maintaining the maximum deflection of the membrane at approximately 3 μm. Figure 15b indicates that the introduction of the overload-protection column significantly suppresses the increase in stress in the piezoelectric layer. However, the overload-protection capacity is not unlimited. As shown in Figure 15c, after contact occurs, the maximum stress *σ_max_* in the overload-protection column continues to increase with the water depth, with the maximum protection depth reaching 382 m.

Based on the optimized *h*–*r* design structure obtained from the simulations, the maximum operating depth of the hydrophone can be determined by analyzing the stress response characteristics of the overload-protection column under various working depths. Two criteria must be satisfied:

- The first: the column must maintain structural stability. As described in Section 4, its maximum stress must satisfy $\sigma_{max}\leq 70\;MPa$;
- The second: each membrane layer must meet the hydrostatic pressure resistance requirement. As specified in Section 5.1, the maximum radial stress *σ_rmax_* of each layer remains below 1% of its corresponding Young’s modulus.

The final optimized design allows the hydrophone to maintain reliable overload protection at a depth of 382 m, demonstrating that the inclusion of the overload-protection column significantly enhances the device’s durability in extreme operating environments and provides effective assurance for its safe operation.

### 5.3. Effect of the Overload-Protection Column on Hydrophone Sensitivity

**Figure 16:**
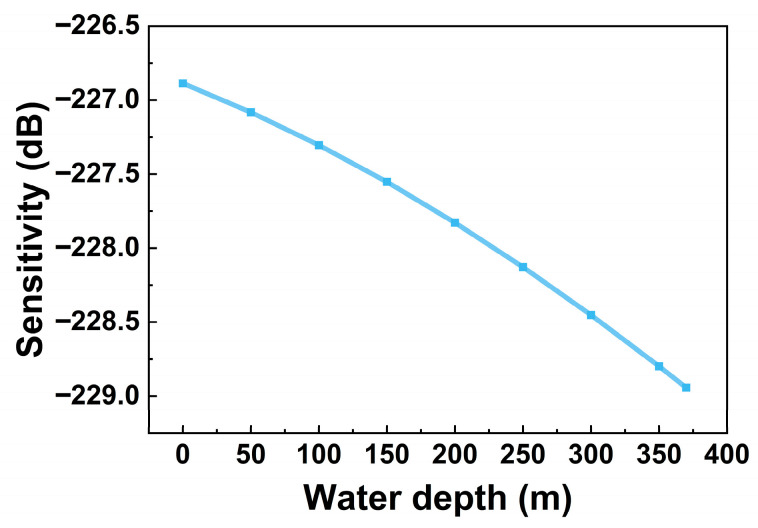
Hydrophone sensitivity at different water depths (@200 Hz, ref. 1 V/μPa).

The variation in the single-membrane sensitivity of the hydrophone with the overload-protection column at different water depths is shown in Figure 16. It can be observed that the sensitivity of the hydrophone gradually decreases as the hydrostatic pressure increases.

**Figure 17:**
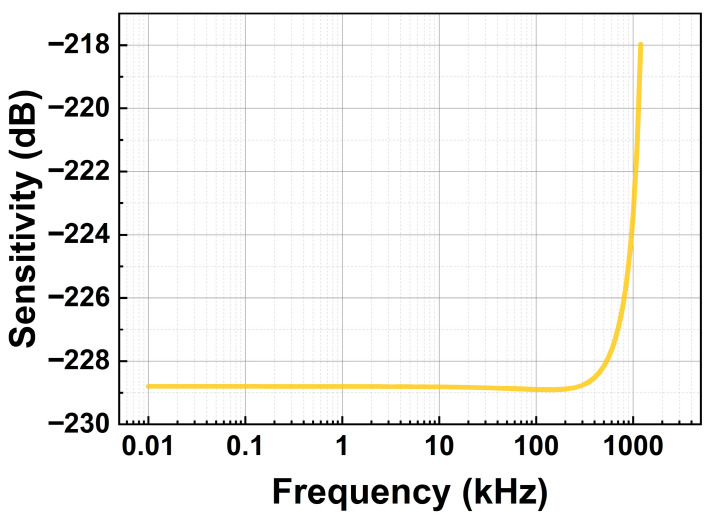
Frequency response of the hydrophone with the overload-protection column (@350 m, ref. 1 V/μPa).

Figure 17 shows the frequency response of the hydrophone with overload-protection columns at a water depth of 350 m, considering the combined effects of back cavity pressure and damping force. As observed, over the frequency range of 10 Hz to 200 kHz, the response remains relatively flat, with a sensitivity of approximately −228.8 dB (@350 m, ref. 1 V/μPa). However, in the higher frequency range of 10 kHz to 200 kHz, the sensitivity decreases slightly due to the increase in damping force *F_d_* with frequency.

**Figure 18:**
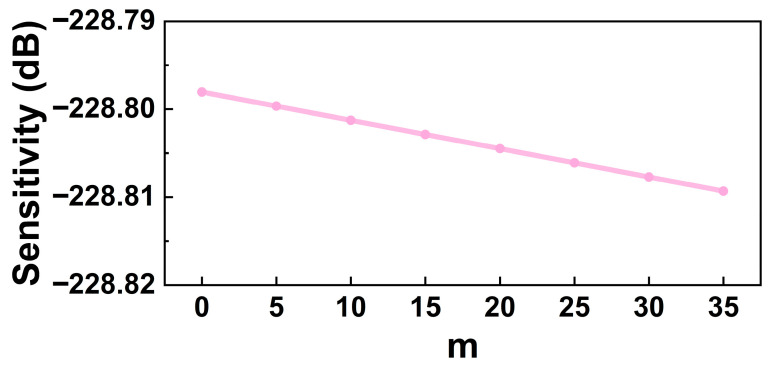
Hydrophone sensitivity for different numbers of overload-protection columns (@350 m, 200 Hz, ref. 1 V/μPa).

The single-membrane sensitivity of the hydrophone for different numbers of overload-protection columns in the cavity is shown in Figure 18. Under the optimal design, the sensitivity decreases slightly as the number of columns increases; however, the overall effect on hydrophone sensitivity remains small. This indicates that, through appropriate design of the *h*–*r* parameters, the influence of back cavity gas on sensitivity can be effectively reduced.

Table 3 compares the performance of similar hydrophones reported in the literature. The results show that, with a single membrane and without amplification circuitry, the hydrophone developed in this study maintains a sensitivity comparable to that of similar devices, while its hydrostatic pressure resistance is significantly improved.

## 6. Conclusions

This study presents an optimization design of overload-protection columns for MEMS hydrophones based on theoretical analysis, finite-element simulations, and fabrication considerations. A comprehensive design methodology is established, and the effects of key geometric parameters on structural performance are analyzed.

The main conclusions are summarized as follows:

1. A small-radius, thick membrane with high flexural stiffness significantly enhances pressure resistance, enabling the membrane to withstand hydrostatic pressures up to 3.6 MPa.
2. Overload-protection columns with intermediate slenderness, having a radius between 5.5 μm and 7.5 μm, satisfy the design requirements.
3. Optimized columns (h=42 μm, r=7.5 μm) effectively suppress membrane deflection and stress growth with minimal impact on sensitivity, increasing the pressure-resistant depth from 360 m to 382 m.
4. Hydrophone sensitivity decreases with increasing hydrostatic pressure, while a relatively flat dynamic response is maintained over 10 Hz–200 kHz. During free vibration (depth < 360 m), the sensitivity is weakly affected by the number of columns, remaining around −228.8 dB (@350 m, 200 Hz, ref. 1 V/μPa).

Overall, the proposed design provides effective guidance for improving pressure resistance while maintaining sensitivity in MEMS piezoelectric hydrophones.

## Figures and Tables

**Table 1:** Material parameters of the MEMS hydrophone membrane with overload-protection column.

Material	Function	Thickness *t* (μm)	Density *ρ* (kg/m^3^)	Young’s Modulus *E* (GPa)	Poisson’s Ratio *ν*
Mo	Top and bottom electrodes	0.2	10,200	312	0.31
AlN	Piezoelectric element	1.0	3300	348.42 [22]	0.24 [22]
SiO_2_	Stress compensation layer	1.0	2200	70	0.17
	Thermally oxidized layer				
Si	Structural support layer	10.0	2329	170	0.28
**Elasticity matrix of AlN (GPa)**		**Coupling matrix of AlN (C/m^2^)**			
$\left[\begin{array}{cccccc} 410 & 149 & 99 & 0 & 0 & 0\\ 149 & 410 & 99 & 0 & 0 & 0\\ 99 & 99 & 389 & 0 & 0 & 0\\ 0 & 0 & 0 & 125 & 0 & 0\\ 0 & 0 & 0 & 0 & 125 & 0\\ 0 & 0 & 0 & 0 & 0 & 130.5 \end{array}\right]$		$\left[\begin{array}{cccccc} 0 & 0 & 0 & 0 & -0.48 & 0\\ 0 & 0 & 0 & -0.48 & 0 & 0\\ -0.58 & -0.58 & 1.55 & 0 & 0 & 0 \end{array}\right]$			

Note: The vibrating membrane radius is 200 μm, and the top electrode radius is 150 μm.

**Table 2:** Maximum radial stress *σ_rmax_* of each membrane layer at a water depth of 375 m.

Membrane Layer	Maximum Radial Stress *σ_rmax_* (MPa)	Maximum Allowable Radial Stress (MPa)
Top electrode (Mo)	254.87	3120
AlN layer	1053.03	3484.2
Bottom electrode (Mo)	741.65	3120
SiO_2_ layer	148.94	700
Device Si layer	406.91	1700
Buried oxide layer (SiO_2_)	177.08	700

**Table 3:** Performance comparison of similar hydrophones.

Works	Material	Performance-Enhancing Structure	Sensitivity (Ref. 1 V/μPa)	Pressure Resistance
Mikinori et al. [30]	PZT	Thickness vibration	−243 dB	Good but without specific data
Choi et al. [12,14]	PZT	Pressure-balancing module	−227.5 dB −215 dB	1.5 MPa 0.78 MPa
Xu et al. [16,17]	AlN	Torus columns 10×10 cell array design	−180 dB (with 40 dB gain)	1 MPa
Huang et al. [6]	Sc-AlN	Separated-electrode design	−166.8 dB (with 40 dB gain)	>1 MPa
This work	AlN	High bending stiffness design Overload-protection columns	−228.8 dB (@350 m, 200 Hz)	3.82 MPa

## Data Availability

The original contributions presented in this study are included in the article. Further inquiries can be directed to the corresponding authors.

## Nomenclature

The following symbols are mainly used in this manuscript:

*A*	Cross-sectional area of the column (μm^2^)
*a*, *b*	Material constants (MPa)
*c*	Squeeze-film damping coefficient (N·s/m)
*D*	Equivalent flexural rigidity of the composite membrane (N·m)
*E*	Elastic modulus (GPa)
*F_d_*	Damping force (N)
*f*_0_	First-order resonant frequency of the membrane (kHz)
*H*	Back cavity height (μm)
*h*	Overload-protection column height (μm)
*h*_0_	Gap height (μm)
*I*	Area moment of inertia of the column (μm^4^)
*i*	Radius of gyration of the column (μm)
*k_p_*, *k_m_*	Axial compressive stiffness of the column and local stiffness of the membrane (N/m)
*M*	Membrane mass (kg)
*m*	Number of the overload-protection column
*N*, *k*	Number of layers in the composite membrane and the index assigned to each layer
*n_st_*	Safety factor
*P*_0_, *P*_1_, *P_atm_*	Initial pressure and the compressed pressure (Pa)
*P_atm_*_,_ *P_water_*	Standard atmospheric pressure and water pressure (Pa)
*Q*	Mechanical quality factor
*R*	Radius of the membrane (μm)
*r*	Overload-protection column radius (μm)
*S*	Effective area between the membrane and the substrate (μm^2^)
*t*	Thickness of each membrane material layer (μm)
*V*_0_, Δ*V*, *V*_1_	Initial effective volume, change volume and compressed volume of the cavity (μm^3^)
*v*	Vibration velocity of the membrane (m/s)
*x*	Maximum deflection of the vibrating membrane (μm)
*z*, *z*^0^	Surface coordinates of each layer and neutral plane coordinate (μm)
*η*	Gas viscosity
*λ*	Slenderness ratio of the overload-protection column
*λ_P_*, *λ*_0_	Material constants for distinguishing column slenderness regimes
*μ*	Effective length coefficient of the column
*ν*	Poisson’s ratio of each membrane material
*ρ*	Density of each membrane material (kg/m^3^)
*ρ_S_*	Areal density of the composite membrane (kg/m^2^)
*σ_cr_*	Theoretical critical stress of the overload-protection column (MPa)
*σ_P_*, *σ*_0_	Proportional limit and ultimate stress of the material (MPa)
*σ_s_*, *σ_bc_*, *σ_bt_*	Yield strength of plastic materials, ultimate compressive strength and ultimate tensile strength of brittle materials (MPa)
*σ_avg_*, *σ_max_*	Average and maximum stress of the overload-protection column (MPa)
*σ_cr_*_0_	Actual critical stress of the overload-protection column (MPa)
*σ_cr-E_*	Theoretical critical stress of the column determined based on Euler’s formula (MPa)
[*σ*], [*σ*_0_]	Theoretical allowable stress and actual allowable stress of the column (MPa)
*σ_rmax_*	Maximum radial stress of each layer (MPa)
*ω*_0_	Resonant angular frequency of the membrane (rad/s)
